# Association between the Achievement of Target Range CKD-MBD Markers and Mortality in Prevalent Hemodialysis Patients in Taiwan by Using the Kidney Disease: Improving Global Outcomes Clinical Guidelines

**DOI:** 10.1155/2016/1523124

**Published:** 2016-11-27

**Authors:** Ying Liu, Wen-Chin Lee, Ben-Chung Cheng, Lung-Chih Li, Chih-Hsiung Lee, Wen-Xiu Chang, Jin-Bor Chen

**Affiliations:** ^1^Department of Nephrology, Tianjin First Center Hospital, Tianjin, China; ^2^Division of Nephrology, Department of Internal Medicine, Kaohsiung Chang Gung Memorial Hospital, Chang Gung University College of Medicine, Taoyuan, Taiwan

## Abstract

*Background. *This study evaluated the association between achieving target chronic kidney disease-mineral and bone disorder (CKD-MBD) marker levels and mortality in Taiwanese hemodialysis (HD) patients. Target levels were based on the Kidney Disease: Improving Global Outcomes (KDIGO) guidelines.* Methods.* We performed a retrospective medical record review of 1126 HD patients between 2009 and 2013. A logistic regression model was used to evaluate the relationship between achieving target marker levels and the risk for all-cause and cardiovascular (CV) mortality. Reference target ranges were 7.9 ≤ calcium (Ca) ≤ 9.9 mg/dL, 2.4 ≤ phosphate (P) ≤ 4.7 mg/dL, and 144 ≤ intact parathyroid hormone (iPTH) ≤ 648 pg/mL.* Results.* Achievement of target P levels was associated with a lower risk for all-cause mortality compared to achievement of either target Ca or iPTH levels. Achieving target P + iPTH levels (OR 1.32) was associated with a lower odds ratio for all-cause mortality compared to achieving target Ca + P (OR 1.66) and Ca + iPTH (OR 1.43) levels. Similar trends were observed for CV mortality risk.* Conclusions.* The present study demonstrated that achieving serum P levels within the KDIGO target range is the most important factor for lowering mortality in HD patients.

## 1. Introduction

Chronic kidney disease-mineral and bone disorder (CKD-MBD) has emerged as an important factor in the care of CKD patients. The Kidney Disease: Improving Global Outcomes (KDIGO) guidelines define CKD-MBD as a systemic disorder of mineral and bone metabolism due to CKD and manifested by either one or a combination of the following: (1) abnormalities of calcium (Ca), phosphorus (P), parathyroid hormone (PTH), or vitamin D metabolism; (2) abnormalities in bone turnover, mineralization, volume, linear growth, or strength; and (3) vascular or other soft-tissue calcification [1]. In the past several decades, many epidemiologic studies have revealed that CKD-MBD markers are associated with higher rates for both all-cause and cardiovascular (CV) mortality [2–7]. Accordingly, clinical guidelines have set the target range for three CKD-MBD markers (namely, Ca, P, and PTH) in CKD-MBD management. The two clinical guidelines adopted by the majority of clinicians are the US National Kidney Foundation Kidney Disease Outcomes Quality Initiative (NFK KDOQI) guidelines implemented in 2003 [8] and the Kidney Disease: Improving Global Outcomes (KDIGO) guidelines implemented in 2009 [1]. Owing to variations in health support systems and living habits around the world, discrepancies between target ranges and achievement rates in CKD-MBD markers have been reported [9–12]. In the Dialysis Outcomes and Practice Patterns Study, only 4.6% to 5.5% of patients were within range for all four CKD-MBD markers (serum P, albumin-corrected Ca, Ca-P product, and iPTH), and only 23% to 28% patients fell within the guidelines for at least three markers [9]. Fouque et al. [13] investigated the CKD-MBD marker targets in the KDIGO guidelines by using data from the French Phosphate and Calcium Observatory Study and found low achievement rates for Ca (59.4%), P (32.6%), and PTH (60.7%) targets and an extremely low rate of target achievement for all three markers (12.3%). Another report from Chinese dialysis patients also revealed suboptimal achievement rates based on the NFK KDOQI clinical guidelines for target Ca (38.6%), P (37.6%), and PTH (26.5%) levels [14].

The NFK KDOQI guidelines for CKD-MBD recommend that clinicians maintain serum Ca, P, and iPTH levels within a narrow range [8]. In contrast, the KDIGO CKD-MBD guidelines recommend maintaining serum Ca, P, and iPTH levels in a reference range determined by individual laboratory tests [1]. However, the recommendations of these two clinical guidelines are primarily based on retrospective observational studies. Therefore, the ability of these recommendations to predict mortality and morbidity across time and countries has subsequently been challenged. To date, there have been no randomized controlled trials which have determined the ideal ranges for CKD-MBD markers, and so we can only rely on the results from epidemiological observational studies.

As the largest hospital-facilitated HD center in Taiwan, we evaluated the relationship between achievement rates of KDIGO determined CKD-MBD marker levels and mortality between 2009 and 2013.

## 2. Materials and Methods

### 2.1. Study Participants

Patients who received regular outpatient HD three times per week at Kaohsiung Chang Gung Memorial Hospital in Taiwan were enrolled in the study. These patients were tracked from January 1, 2009, until December 31, 2013. In total, the medical records of 1126 HD patients were reviewed and the demographic data and CKD-MBD laboratory marker levels were compiled. Patients were excluded if (1) they did not receive regular HD thrice weekly and/or (2) they did not receive continuous HD for at least 3 months in the study hospital.

### 2.2. Retrospective Analysis of Patient Medical Records

We retrospectively analyzed serial hemogram results and biochemical data for each patient between January 2009 and December 2013. Baseline characteristics included the baseline levels of blood albumin, hemoglobin (Hb), cholesterol, triglyceride, fasting blood glucose, ferritin, intact PTH (iPTH), noncorrected Ca, P, potassium (K), uric acid, *Kt*/*V* urea score, and cardiothoracic (CT) ratio. Laboratory values for blood analysis were measured monthly with the exceptions of ferritin (measured every 3 months), iPTH (measured every 6 months), and *Kt*/*V* urea score (Daugirdas method) (measured every 6 months) [15]. Blood sampling was performed in the mid-week session before routine hemodialysis (Wednesday, Thursday). *Kt*/*V* scores were calculated using the following equation: *Kt*/*V* urea score = −ln⁡(*R* − 0.008 × *t*) + [4 − (3.5 × *R*)] × UF/*W*, where *R* is the ratio of postdialysis BUN to predialysis BUN, *t* (in hours) is the duration of dialysis, UF (L) is the ultrafiltrate amount, and *W* (kg) is the postdialysis body weight. The reference values in our laboratory for CKD-MBD markers are as follows: serum Ca 7.9–9.7 mg/dL, serum P 2.4–4.7 mg/dL, and serum iPTH 14–72 pg/mL. All blood samples were analyzed using commercial kits and an autoanalyzer (Hitachi 7600-210, Hitachi Ltd., Tokyo, Japan). Albumin was measured by the bromocresol green (BCG) method. iPTH was measured by chemiluminescence immunoassay (Siemens Healthcare Diagnostics Inc., USA). To determine the cardiothoracic (CT) ratio for each patient, chest radiography was performed following HD. Cardiac size was measured as the distance between parallel lines drawn down to the most lateral points on both sides of the heart. Thoracic width was measured as the distance between parallel lines drawn down the inner aspect of the widest points of the rib cage. The CT ratio was defined as cardiac size/thoracic width. All patients received HD with dialyzers that had an effective surface area > 2.0 m^2^. Majority subjects received dialysate Ca 3.0 mEq/L. Dialysate Ca 3.5 mEq/L or 2.5 mEq/L was used when the patients presented with hypocalcemia or hypercalcemia in the routine monthly blood examinations.

### 2.3. Approval

The study protocol was approved by the Committee on Human Research at Kaohsiung Chang Gung Memorial Hospital (101-1595B) and was conducted in accordance with the Declaration of Helsinki (1964). The requirement for obtaining informed consent from study patients was waived in accordance with the retrospective data review regulations of the Committee on Human Research at Kaohsiung Chang Gung Memorial Hospital.

### 2.4. Patient Demographics and Baseline Characteristics

The demographics and baseline characteristics of the study population are represented as numbers and percentages for categorical data and as means ± standard deviation for continuous data. Demographics included age, sex, HD vintage, etiology of renal failure, erythropoietin (EPO) use, vitamin D analogs use, antihypertensive agent use, iron use, and parathyroidectomy status. The study endpoints were all-cause and cardiovascular (CV) mortality.

### 2.5. Mortality versus Patient Responses according to KDIGO Guidelines (Logistic Model)

According to the KDIGO clinical practice guidelines, patients who achieved targeted levels of serum Ca (7.9–9.9 mg/dL) during the study constituted the target response group. For P levels, “normal values of laboratory” according to KDIGO recommended values were 2.4–4.7 mg/dL in our HD center. For iPTH levels, the target response range was 144–648 pg/mL. We calculated the mean values of CKD-MBD markers (Ca, P) in each study subject in the first three continuous months when the subjects were enrolled for observation. iPTH levels were measured every six months and the first three values were used for mean calculation. Then the mean values were used for odds ratio (OR) analysis. A logistic regression model was used to demonstrate the association between the risk for mortality and the achievement of the target response range for corrected Ca, P, and iPTH levels in the study population. All of the patients in the target response group were defined as the reference group. Crude and adjusted OR with corresponding 95% confidence intervals (CI) were calculated using logistic regression analysis. Two categories of covariates were considered: patient demographics and baseline characteristics. A *p* value < 0.05 was considered statistically significant. All statistical analyses were performed using SAS 9.3.

## 3. Results

### 3.1. Patient Demographics and Baseline Characteristics

A total of 1126 HD patients were enrolled in the study. The mean duration of follow-up was 3.38 ± 1.08 years. The mean age of the study population was 60.0 ± 12.4 years old, and the mean HD vintage was 5.74 ± 5.34 years. Over the 5-year observation period, 240 patients experienced all-cause mortality, and 48 patients experienced CV mortality (Table 1). Additional patient characteristics, including laboratory parameters at baseline, HD adequacy indices, and CT ratios, are shown in Table 2.

### 3.2. Logistic Regression Analysis of Risk of Mortality versus Patient Responses according to KDIGO Guidelines

We examined the relationship between achieving target levels of three separate CKD-MBD markers (Ca, P, and iPTH) according to the KDIGO clinical guidelines and the risk for all-cause and CV mortality over the five-year study interval. The unadjusted logistic regression model demonstrated that hypocalcemia (<7.9 mg/dL) was significantly associated with a higher OR (OR 2.41, 95% CI 1.22–4.77, and *p* = 0.01) for all-cause mortality, and the trend still appeared after adjusted model analysis (models 2 and 3) (Table 3). There were also no significant differences between Ca levels and CV mortality by multivariable adjusted analysis. Hyperphosphatemia (P > 4.7 mg/dL) was significantly associated with an increased OR for all-cause and CV mortality according to multivariable adjusted logistic regression models (OR 1.82, 95% CI 1.17–2.82, and *p* = 0.007; OR 2.62, 95% CI 1.19–5.76, and *p* = 0.01, resp.) (Table 4). Using an iPTH reference range of 144–648 pg/mL, all three models demonstrated that low (<144 pg/mL) iPTH levels were significantly associated with higher OR for all-cause mortality, but not for CV mortality. High (>648 pg/mL) iPTH levels were not significantly associated with higher OR for all-cause and CV mortalities by multivariable adjusted analysis (Table 5).

We also examined the relationship between simultaneously achieving target levels for all three CKD-MBD markers (Ca, P, and iPTH) according to the KDIGO clinical guidelines and the risk for mortality. The reference group included patients who achieved targets simultaneously for all three CKD-MBD markers (Table 6). Patients who achieved target Ca levels had a lower risk for all-cause mortality compared to those who only achieved target P or iPTH levels by multivariable adjusted models. Multivariable adjusted model analyses of the risk for mortality associated with simultaneously achieving two target CKD-MBD marker levels revealed that achieving target P and iPTH (P + iPTH) levels (OR 1.32) was associated with a lower OR for all-cause mortality compared to achieving Ca + P (OR 1.66) and Ca + iPTH (OR 1.43) levels. Achieving target Ca + P levels demonstrated the highest OR (compared to Ca + iPTH and P + iPTH) for all-cause mortality in all three logistic regression models. Analysis of the risk of CV mortality revealed similar trends, where achieving target P + iPTH levels showed the lowest OR compared to achieving target levels for any other combination of CKD-MBD markers. Patients who did not achieve any of the three-target CKD-MBD marker levels demonstrated increased risk for all-cause mortality in all three logistic regression models, compared to patients who achieved any two-target CKD-MBD marker levels. In contrast, this trend was not apparent for risk of CV mortality according to case-mix adjusted and multivariable adjusted models (Table 6).

Patients number in each achievement of CKD-MBD markers was shown in supplementary Tables  1 and 2 in Supplementary Material available online at http://dx.doi.org/10.1155/2016/1523124.

## 4. Discussion

In the present study, we examined the association between mortality and achievement of KDIGO recommended target ranges of CKD-MBD markers (Ca, P, and iPTH) in a cohort of HD patients. Compared to reference serum Ca levels (7.9–9.9 mg/dL), serum Ca levels < 7.9 mg/dL and >9.9 mg/dL were associated with an increased risk for all-cause mortality in all three logistic regression models (unadjusted, case-mix adjusted, and multivariable-adjusted). However, only patients with Ca < 7.9 mg/dL in the three models yielded a statistically significant increase in the risk for all-cause mortality. Our results are in slight disagreement with those previously reported by Fouque et al. who demonstrated a U-shape relationship between HR for mortality and total serum Ca concentrations [13]. We suppose the different patient number recruitment and study design may contribute this variable results. Of note, there have been no randomized controlled trials that have determined the ideal ranges for CKD-MBD markers. A further study in the future is needed.

When we applied KDIGO recommended serum P levels to logistical regression analyses of P level-associated mortality risk, serum P levels < 2.4 mg/dL and > 4.7 mg/dL were generally associated with an increased risk for both all-cause and CV mortality. By multivariable adjusted analysis, we found that serum P levels > 4.7 mg/dL had a significantly increased OR for all-cause and CV mortality. In previous reports, risk for mortality was associated with increased P levels in CKD patients [2, 3, 5, 6]. However, these studies differed from ours with respect to study design and patient population and thus used different cut-off P levels for mortality. The present study enrolled a relatively small number of patients and did not stratify serum P levels extensively, and thus a cut-off serum P level for highest risk for mortality cannot be determined from the present study. Nevertheless, the trend for the association between mortality and high serum P levels demonstrated in this study was similar to previous reports. The present study did not find an association between serum P < 2.4 mg/dL and mortality. Low P levels (≤3.5 mg/dL) at baseline were found to be significantly associated with higher rates for CV mortality in a 3-year HD cohort study in Japan [16]. Another report from a French HD cohort failed to demonstrate a significant association between low P levels (≤2.79 mg/dL) at baseline and mortality over 30 months of follow-up [13]. A report from the United Kingdom demonstrated a lower hazard ratio for all-cause mortality in incident dialysis patients with serum P levels < 3.5 mg/dL [12]. In the HEMO study, lower levels of serum P (≤4.0 mg/dL) did not increase the hazard ratio for all-cause mortality [17]. These inconsistencies highlight the need for further studies in order to clarify the association between low serum P levels and mortality in dialysis patients.

As previously established, high iPTH levels were associated with an increased risk for mortality in HD patients. A J-curve association between PTH levels and risk for mortality has previously been reported [4, 7, 16, 18]. We evaluated the association between KDIGO recommended serum iPTH levels (144–648 pg/mL based on our laboratory test) and risk for all-cause and CV mortality. We found that serum iPTH levels < 144 pg/mL and >648 pg/mL were significantly associated with an increased risk for all-cause mortality, but not for CV mortality. This result is different from the result previously reported by Fouque et al. which did not find a J-curve relationship between iPTH levels and mortality risk [13]. According to the KDIGO recommended target range for serum iPTH levels, iPTH levels > 585 pg/mL were not significantly associated with an increased risk for mortality. The authors attributed this finding to the fact that relatively few patients with serum iPTH levels exceeding 700 pg/mL were included in the study [13]. Our study failed to demonstrate a positive association between high serum iPTH levels and CV mortality in a sample of 1126 patients, and it is possible that this finding may be due to the relatively small sample size analyzed.

We also examined the relationship between achieving KDIGO determined target levels of all three CKD-MBD markers and the risk for all-cause and CV mortality. Case-mix adjusted and multivariable adjusted logistic regression analyses revealed that patients who achieved target serum Ca levels demonstrated a lower risk for all-cause mortality compared to those who achieved target serum P or iPTH levels. When examining the association between simultaneously achieving target levels for two CKD-MBD markers and the risk for mortality, we found that patients who achieved target P + iPTH levels had a lower risk for all-cause and CV mortality than those who achieved either Ca + P or Ca + iPTH targets by fully adjusted model analysis. Based on this finding, we suggest that achieving target serum Ca level or simultaneously achieving P + iPTH levels should be prioritized in the management of CKD-MBD in prevalent HD patients.

Although these results provide valuable CKD-MBD management data obtained over a long period of 5 years, the present study still has some limitations. First, the study was retrospective and the study population was limited to patients in a single HD center. Thus, center-specific effects cannot be avoided. Second, CKD-MBD care was managed by multiple nephrologists in one center. Thus, differences in clinical practice among the different nephrologists could have resulted in heterogeneous CKD-MBD management. Unfortunately, the impact of this limitation on the clinical analysis performed in the present study cannot be addressed. Third, the sample size was relatively small, and other traditional and/or nutritional factors that may influence survival in HD patients were not evaluated in the present study. Finally, the effects of different drugs (e.g., cinacalcet or P-binders such as lanthanum carbonate, sevelamer hydrochloride, and sevelamer carbonate) on HD patient survival were not evaluated. However, these drugs are not covered by medical reimbursement schemes in Taiwan, and thus their use in Taiwanese HD patients is limited. Furthermore, we believe that these drugs would not have had a significant influence on the results presented in this study.

## 5. Conclusions

The present study provides valuable data on the management of CKD-MBD in HD patients followed up at one HD center over a five-year period. The results presented herein, with regard to the association between different CKD-MBD markers and the risk for all-cause and CV mortality in prevalent HD patients, are generally accordant with those cited by previous epidemiological studies. When applying KDIGO recommended target ranges for CKD-MBD markers, the present study demonstrated that patients who achieved the target range for serum Ca levels had lower risk for mortality compared to those who achieved the target range for serum P or iPTH levels. Furthermore, patients who achieved the target ranges for both P and iPTH levels demonstrated a significantly lower risk for mortality compared to those who achieved target ranges for Ca and P levels or Ca and iPTH levels.

## Supplementary Material

Patients number in each achievement of CKD-MBD markers was shown in supplementary Tables 1 and 2.

## Figures and Tables

**Table 1:** Summary of demographics.

Variable	Subjects
	*N* = 1126
Duration of follow-up (year)	3.38 ± 1.08
Age (year)	60.0 ± 12.4
Gender	
Male	535 (47.51%)
Female	591 (52.49%)
Hemodialysis vintage (year)	5.74 ± 5.34
Etiology of renal failure	
Glomerulonephritis	174 (15.45%)
Diabetes mellitus	343 (30.46%)
Others	609 (54.09%)
EPO use	
Yes	802 (90.62%)
No	83 (9.38%)
Missing	241
Vitamin D analogs use	
Yes	237 (21.68%)
No	856 (78.32%)
Missing	33
Antihypertensive agent use	
Yes	382 (34.98%)
No	710 (65.02%)
Missing	34
Iron use	
Yes	103 (9.43%)
No	989 (90.57%)
Missing	34
Parathyroidectomy	
Yes	188 (17.12%)
No	910 (82.88%)
Missing	28
All-cause mortality	
Yes	240 (21.31%)
Cardiopulmonary system	72 (6.39%)
Central nervous system	22 (1.95%)
Infection	52 (4.62%)
Gastrointestinal hepatobiliary system	28 (2.49%)
Malignant tumor	26 (2.31%)
Other	40 (3.55%)
No	886 (78.69%)
Cardiovascular mortality	
Yes	48 (4.26%)
No	1078 (95.74%)

**Table 2:** Summary of laboratory parameters.

Variable	Subjects
	*N* = 1126
Albumin (gm/dL)	
*N*	1091
Mean ± SD	3.84 ± 0.34
Hb (g/dL)	
*N*	1091
Mean ± SD	10.53 ± 1.36
Cholesterol (mg/dL)	
*N*	1089
Mean ± SD	176.04 ± 40.57
Triglyceride (mg/dL)	
*N*	1088
Mean ± SD	165.70 ± 130.11
Glucose (fasting) (mg/dL)	
*N*	1084
Mean ± SD	148.92 ± 72.03
Ferritin (ng/mL)	
*N*	1083
Mean ± SD	495.60 ± 417.86
iPTH (pg/mL)	
*N*	1072
Mean ± SD	355.98 ± 451.77
Ca (mg/dL)	
*N*	1090
Mean ± SD	9.14 ± 0.86
P (mg/dL)	
*N*	1091
Mean ± SD	4.88 ± 1.51
K (meq/L)	
*N*	1091
Mean ± SD	4.95 ± 0.76
Uric acid (mg/dL)	
*N*	1081
Mean ± SD	7.09 ± 1.41
Urea reduction ratio	
*N*	1090
Mean ± SD	0.74 ± 0.07
*Kt*/*V* Daugirdas	
*N*	1088
Mean ± SD	1.69 ± 0.65
Cardiac/thoracic ratio	
*N*	1056
Mean ± SD	0.50 ± 0.08

Hb, hemoglobin; iPTH, intact parathyroid hormone.

**Table 3:** Odds ratio of all-cause and cardiovascular (CV) mortality by achievement of KDIGO clinical guideline, Ca target.

	OR (95% CI)					
	Model 1		Model 2		Model 3	
Ca (mg/dL), all-cause						
<7.9	2.41 (1.22~4.77)	0.01	3.24 (1.19~8.80)	0.02	7.11 (1.25~40.3)	0.02
7.9~9.9	1.00		1.00		1.00	
>9.9	1.51 (0.92~2.48)	0.10	1.56 (0.88~2.77)	0.13	1.46 (0.77~2.78)	0.24

Ca (mg/dL), CV						
<7.9	2.02 (0.60~6.83)	0.25	0.67 (0.08~5.73)	0.71	0.71 (0.06~8.95)	0.78
7.9~9.9	1.00		1.00		1.00	
>9.9	0.82 (0.25~2.69)	0.73	1.01 (0.28~3.58)	0.99	0.89 (0.23~3.47)	0.86

Logistic regression model:

Model 1: unadjusted.

Model 2: adjusted for age, sex, hemodialysis vintage, etiology of renal failure, EPO, vitamin-D analogs, antihypertensive agent, iron use, and parathyroidectomy.

Model 3: model 2 + baseline laboratory results (albumin, hemoglobin, cholesterol, triglyceride, glucose (fasting), ferritin, P, iPTH, potassium, uric acid, *Kt*/*V* urea, and cardiac-thoracic ratio).

**Table 4:** Odds ratio of all-cause and CV mortality by achievement of KDIGO clinical guideline, P target.

	OR (95% CI)					
	Model 1		Model 2		Model 3	
P (mg/dL), all-cause						
<2.4	1.61 (0.80~3.22)	0.17	1.16 (0.42~3.17)	0.77	0.89 (0.18~4.35)	0.88
2.4~4.7	1.00		1.00		1.00	
>4.7	1.13 (0.82~1.55)	0.47	1.76 (1.19~2.60)	0.004	1.82 (1.17~2.82)	0.007

P (mg/dL), CV						
<2.4	2.13 (0.62~7.31)	0.23	1.78 (0.36~8.88)	0.48	4.70 (0.69~31.9)	0.11
2.4~4.7	1.00		1.00		1.00	
>4.7	1.60 (0.86~2.97)	0.13	2.59 (1.27~5.32)	0.009	2.62 (1.19~5.76)	0.01

Logistic regression model:

Model 1: unadjusted.

Model 2: adjusted for age, sex, hemodialysis vintage, etiology of renal failure, EPO, vitamin-D analogs, antihypertensive agent, iron use, and parathyroidectomy.

Model 3: model 2 + baseline laboratory results (albumin, hemoglobin, cholesterol, triglyceride, glucose (fasting), ferritin, Ca, iPTH, potassium, uric acid, *Kt*/*V* urea, and cardiac-thoracic ratio).

**Table 5:** Odds ratio of all-cause and CV mortality by achievement of KDIGO clinical guideline, iPTH target.

	OR (95% CI)					
	Model 1		Model 2		Model 3	
iPTH (pg/mL), all-cause						
<144	1.70 (1.25~2.33)	<0.001	2.00 (1.31~3.05)	0.001	1.95 (1.21~3.14)	0.006
144~648	1.00		1.00		1.00	
>648	1.67 (1.03~2.70)	0.03	2.14 (1.21~3.77)	0.008	1.47 (0.77~2.81)	0.24

iPTH (pg/mL), CV						
<144	1.12 (0.60~2.12)	0.72	1.20 (0.54~2.70)	0.65	1.68 (0.70~4.02)	0.24
144~648	1.00		1.00		1.00	
>648	0.69 (0.21~2.31)	0.54	1.00 (0.28~3.54)	0.99	0.54 (0.13~2.21)	0.39

Logistic regression model:

Model 1: unadjusted.

Model 2: adjusted for age, sex, hemodialysis vintage, etiology of renal failure, EPO, vitamin-D analogs, antihypertensive agent, iron use, and parathyroidectomy.

Model 3: model 2 + baseline laboratory results (albumin, hemoglobin, cholesterol, triglyceride, glucose (fasting), ferritin, Ca, P, potassium, uric acid, *Kt*/*V* urea, and cardiac-thoracic ratio).

**Table 6:** Odds ratio of all-cause and CV mortality by achievement of KDIGO clinical guideline.

	OR (95% CI)					
	Model 1		Model 2		Model 3	
All-cause						
All	1.00		1.00		1.00	
Ca + P	1.52 (1.05~2.21)	0.02	1.85 (1.16~2.95)	0.009	1.66 (1.01~2.73)	0.04
Ca + iPTH	0.96 (0.60~1.52)	0.84	1.39 (0.81~2.39)	0.23	1.43 (0.77~2.68)	0.25
P + iPTH	1.07 (0.46~2.52)	0.87	1.50 (0.59~3.78)	0.39	1.32 (0.46~3.77)	0.60
Ca	1.85 (1.14~3.00)	0.01	4.02 (2.12~7.63)	<0.001	3.16 (1.52~6.59)	0.002
P	3.95 (1.65~9.46)	0.002	4.54 (1.61~12.8)	0.004	3.55 (1.05~12.0)	0.04
iPTH	2.51 (1.08~5.83)	0.03	4.69 (1.56~14.1)	0.005	4.70 (1.44~15.4)	0.01
None	2.19 (1.06~4.52)	0.03	1.99 (0.77~5.13)	0.15	3.91 (1.00~15.3)	0.05

CV						
All	1.00		1.00		1.00	
Ca + P	1.30 (0.60~2.85)	0.50	1.30 (0.49~3.41)	0.59	1.39 (0.51~3.77)	0.51
Ca + iPTH	1.75 (0.78~3.92)	0.17	2.58 (1.04~6.39)	0.04	2.96 (1.08~8.13)	0.03
P + iPTH	0.77 (0.10~5.96)	0.80	1.40 (0.17~11.9)	0.75	1.01 (0.11~9.66)	0.99
Ca	1.39 (0.50~3.89)	0.52	4.13 (1.32~13.0)	0.01	4.35 (1.20~15.9)	0.02
P	NA		NA		NA	
iPTH	3.71 (1.01~13.6)	0.04	3.47 (0.62~19.4)	0.15	2.40 (0.40~14.3)	0.33
None	1.58 (0.35~7.14)	0.55	0.82 (0.10~6.86)	0.85	1.43 (0.12~16.9)	0.77

Logistic regression model:

Model 1: unadjusted.

Model 2: adjusted for age, sex, hemodialysis vintage, etiology of renal failure, EPO, vitamin-D analogs, antihypertensive agent, iron use, and parathyroidectomy.

Model 3: model 2 + baseline laboratory results (albumin, hemoglobin, cholesterol, triglyceride, glucose (fasting), ferritin, Ca, P, iPTH, potassium, uric acid, *Kt*/*V* urea, and cardiac-thoracic ratio).
