# Supplementary material for: Association between the Achievement of Target Range CKD-MBD Markers and Mortality in Prevalent Hemodialysis Patients in Taiwan by Using the Kidney Disease: Improving Global Outcomes Clinical Guidelines
Source: Biomed Res Int. 2016 Nov 27;2016:1523124. doi: 10.1155/2016/1523124 (PMC5149608; doi:10.1155/2016/1523124)
Supplement: Supplementary file 1 — Patients number in each achievement of CKD-MBD markers was shown in supplementary Tables 1 and 2. [file 1523124.f1.pdf]

Supplementary Table 1. All-cause and CV mortality of each level by achievement of KDIGO clinical guideline - Ca, P and iPTH targets

		All-cause		CV	
	Sample	Event	Percent	Event	Percent
Total	1126	240	21.31%	48	4.26%
Ca (mg/dl)					
< 7.9	37	14	37.84%	3	8.11%
7.9~9.9	1002	202	20.16%	42	4.19%
> 9.9	87	24	27.59%	3	3.45%
P (mg/dl)					
< 2.4	41	12	29.27%	3	7.32%
2.4~4.7	782	160	20.46%	28	3.58%
> 4.7	303	68	22.44%	17	5.61%
iPTH (pg/ml)					
< 144	317	86	27.13%	15	4.73%
144~648	708	127	17.94%	30	4.24%
> 648	101	27	26.73%	3	2.97%

Supplementary Table 2. All-cause and CV mortality of each level by achievement of KDIGO clinical guideline

		All-cause		CV	
	Sample	Event	Percent	Event	Percent
Overall	1126	240	21.31%	48	4.26%
All	471	82	17.41%	16	3.40%
Ca+P	251	61	24.30%	11	4.38%
Ca+iPTH	173	29	16.76%	10	5.78%
P+iPTH	38	7	18.42%	1	2.63%
Ca	107	30	28.04%	5	4.67%
P	22	10	45.45%	0	0.00%
iPTH	26	9	34.62%	3	11.54%
None	38	12	31.58%	2	5.26%
